# The visual coupling between neighbours explains local interactions underlying human ‘flocking'

**DOI:** 10.1098/rspb.2021.2089

**Published:** 2022-03-09

**Authors:** Gregory C. Dachner, Trenton D. Wirth, Emily Richmond, William H. Warren

**Affiliations:** Department of Cognitive, Linguistic, and Psychological Sciences, Brown University, Providence, RI 02912, USA

**Keywords:** collective behaviour, crowd dynamics, pedestrian dynamics, vision-based model, agent-based model

## Abstract

Patterns of collective motion in bird flocks, fish schools and human crowds are believed to emerge from local interactions between individuals. Most ‘flocking' models attribute these local interactions to hypothetical rules or metaphorical forces and assume an omniscient third-person view of the positions and velocities of all individuals in space. We develop a *visual model* of collective motion in human crowds based on the visual coupling that governs pedestrian interactions from a first-person embedded viewpoint. Specifically, humans control their walking speed and direction by cancelling the average angular velocity and optical expansion/contraction of their neighbours, weighted by visibility (1 − occlusion). We test the model by simulating data from experiments with virtual crowds and real human ‘swarms'. The visual model outperforms our previous omniscient model and explains basic properties of interaction: ‘repulsion' forces reduce to cancelling optical expansion, ‘attraction' forces to cancelling optical contraction and ‘alignment' to cancelling the combination of expansion/contraction and angular velocity. Moreover, the neighbourhood of interaction follows from Euclid's Law of perspective and the geometry of occlusion. We conclude that the local interactions underlying human flocking are a natural consequence of the laws of optics. Similar perceptual principles may apply to collective motion in other species.

## 1. Background

Human crowds exhibit patterns of collective motion in many public settings, from train stations and shopping plazas to—sometimes catastrophically—mass events [1,2]. Similar patterns of coordinated motion are observed in bird flocks, fish schools and animal herds, suggesting that diverse systems obey common principles of self-organization [3,4]. It is generally believed that these global ‘flocking' patterns emerge from local interactions between individuals [3–5]. The crux of the problem thus lies in understanding the nature of the local interactions.

Most models of collective motion ascribe these interactions to hypothetical rules or metaphorical forces, often inspired by physical systems, and assume a third-person view of the positions and velocities of all individuals in space [6,7]. Such phenomenological *omniscient models*—including our own [8]—describe relationships between individuals without offering an underlying mechanism. But humans and animals are embedded within collectives and coupled to their neighbours by perceptual information. Here, we develop a *visual model* of collective motion that explains local interactions in terms of the visual coupling, based on optical variables. Not only does the visual model outperform our previous omniscient model, but basic properties of interaction follow from the laws of optics.

Understanding local interactions involves, first, identifying the *rules of engagement* that govern how an individual responds to a neighbour, and second, characterizing the *neighbourhood of interaction* over which the rules operate and the influences of multiple neighbours are combined. Classical ‘zonal' models [9–11] posit three local rules or forces in concentric zones: (i) *repulsion* from neighbours in a near zone to avoid collisions, (ii) *alignment* with the velocity of neighbours in an intermediate zone to generate common motion, and (iii) *attraction* to neighbours in a far zone to ensure group cohesion. Influences are combined by averaging neighbours within a zone, sometimes weighted by their distance [12,13]. An alignment rule by itself is theoretically sufficient to generate collective motion [14], as is the combination of attraction and repulsion [15]. In humans, the prominent social force model [16,17] assumes attraction and repulsion, successfully simulates key crowd scenarios [18,19], and can generate collective motion under certain boundary conditions [20,21]. However, it does not produce realistic individual trajectories [22] or generalize between situations without re-parameterization [17,23].

The strength of such physics-inspired models is that they capture generic properties of collective motion, yet the same global patterns can be generated by different sets of local rules [5,24]. To decipher the actual rules, researchers have turned to behavioural experiments on local interactions [25–28]. We believe that such a ‘bottom-up' approach should be grounded in the perceptual coupling that actually governs these interactions. The coupling incorporates limits on the field of view and sensory range [10,29] as well as the visibility of individual neighbours [30,31]. Moreover, local interactions strongly depend on the optical information that controls locomotion [32,33]. This insight has inspired recent ‘vision-based' models [34–36], but the effective visual coupling remains to be determined.

**Figure 1.**
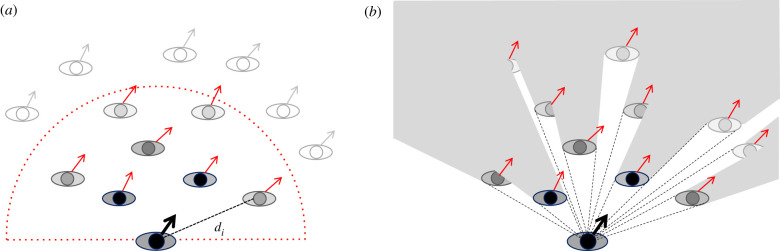
Omniscient and visual models of collective motion. (*a*) *Omniscient model:* a pedestrian (bottom) matches the average heading direction and speed of all neighbours in a 180° neighbourhood. Neighbour weights (grey level) decay exponentially with distance *d_i_* and go to zero at a fixed radius (dotted curve). (*b*) *Visual model:* a pedestrian (bottom) cancels the average angular velocity and optical expansion of all visible neighbours. Neighbour influence decreases with distance owing to Euclid's Law (grey level) and is proportional to neighbour visibility (shaded areas = occluded regions). (Online version in colour.)

We take a bottom-up, experiment-driven approach called ‘behavioural dynamics' [27,37]. Our initial experiments on following in pedestrian dyads [38,39] suggested that humans obey an alignment rule: the follower tends to match the walking direction (*heading*) and speed of the leader. To infer the neighbourhood of interaction, we immersed walking participants in a virtual crowd and manipulated the motions of the avatars; we also analysed observational data on human ‘swarms' [8]. The results showed that pedestrians follow a crowd by averaging the heading directions and speeds of neighbours within a 180° field of view, with weights that decay exponentially with distance to zero around 4 m. The findings led to an omniscient model of collective motion [8] based on the weighted average of neighbour headings and speeds (figure 1*a*; see the electronic supplementary material, equations S1–S4). The model successfully predicts individual trajectories in both virtual crowd experiments and real crowd data [8,40], and the ‘soft metric' neighbourhood generates robust collective motion in simulation [13,41].

Like its predecessors, however, our omniscient model relied on metaphorical forces, assumed physical variables as input, and did not account for the form of the neighbourhood of interaction. In this article, we report new experiments that lead to an embedded visual model (figure 1*b*), predicated on the optical variables that control pedestrian following [42,43]. This new model explains the rules of engagement and the form of the neighbourhood as natural consequences of the laws of optics.

## 2. Experimental methods

### (a) Human subjects

Twelve subjects (seven female, five male) participated in experiment 1, and 10 different subjects (six female, four male) in experiment 2. A power analysis determined that a sample size of eight per experiment was sufficient to achieve a power of 0.85 with *α* = 0.05 and an effect size of 0.5 (*η*^2^ = 0.2) [44]. All participants gave informed consent and were compensated for their time. The research protocol was approved by Brown University's Institutional Review Board in accordance with the principles expressed in the Declaration of Helsinki.

### (b) Equipment

Participants walked freely in a 12 × 14 m tracking area while viewing a virtual environment in a wireless, stereoscopic head-mounted display (HMD, Oculus Rift DK1, 90°H × 65°V field of view, 640 × 800 pixels per eye, 60 Hz refresh rate). Head position and orientation were recorded with an inertial/ultrasonic tracking system (Intersense IS-900; 60 Hz sampling rate) and used to update the display with a latency of 50–67 ms.

### (c) Displays

The virtual environment (WorldViz software) consisted of a green start pole and a grey orientation pole placed 12.73 m apart on a granite-textured ground plane, with a blue sky. The virtual crowd consisted of animated three-dimensional human models (WorldViz Complete Characters). These virtual humans were initially positioned on arcs with the start pole at the centre, at randomly assigned eccentricities (±6°, ±19°, ±32°, ±45°) about the direction to the orientation pole, and then randomly jittered.

### (d) Procedure

To elicit collective motion responses, participants were instructed to ‘walk with the group of virtual humans' and ‘treat them as if they were real people'. On each trial, the participant walked to the start pole and faced the orientation pole. The virtual crowd appeared with their backs to the participant, ‘Begin' was played over headphones, and the crowd began walking forwards (1.0 m s^−1^). After 5 s, the walking direction of some or all virtual humans was perturbed by ±10° (right or left); the display continued for another 7 s and then ‘End' was played. Test trials were preceded by two practice trials to familiarize the participant with walking in a virtual environment.

### (e) Data processing

The time series of head position in the horizontal (*X*–*Y*) plane were low-pass filtered (Matlab) to reduce tracker error and oscillations owing to the gait cycle and then time series of heading direction and walking speed were computed. Left and right perturbation trials were collapsed by multiplying the heading on left-turn trials by −1. The dependent measure was *final heading*, the average heading direction during the last 2 s of each trial. Statistical analyses were performed in Microsoft Excel and JASP. (See the electronic supplementary material for detailed methods.)

## 3. Experiment 1: range of interaction

**Figure 2.**
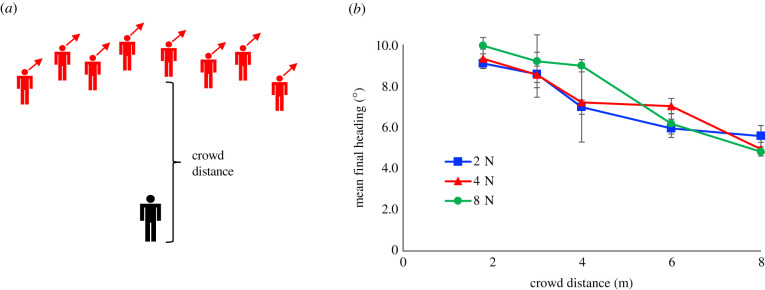
Experiment 1: range of interaction, testing the decay with distance to fully visible neighbours. (*a*) Schematic of virtual crowd, illustrating a rightward heading perturbation. (*b*) Results: mean final heading as a function of crowd distance, for each crowd size (curves). Error bars represent ± s.e.m. (Online version in colour.)

Based on crowd data, the omniscient model holds that neighbour influence decays to zero at a fixed radius of about 4 m [8]. However, it seems likely that interactions with visible neighbours can occur at greater distances. To test the range of interaction, we manipulated the initial distance (1.8, 3.0, 4.0, 6.0 or 8.0 m) of a single row of virtual humans (crowd size 2, 4 or 8), with no occlusion (figure 2*a*). On each trial, their headings were all perturbed in same direction (±10°), and participants were asked to walk with the group.

### (a) Results

We observed a gradual decay in neighbour influence over a much longer distance (figure 2*b*). Final heading decreased from a maximum at 1.8 m (mean *M* = 9.55°) to just half that value at 8 m (*M* = 5.16°) (*F*_4,44_ = 14.93, *p* < 0.001, $\eta_{G}^{2}=$ 0.290). Simple linear extrapolation suggests an interaction range of at least 15 m (*y* = −0.722*x* + 10.8, *r*_14_ *=* −0.95). Consistent with averaging of neighbours, there was no effect of crowd size on final heading (*F*_2,22_ = 0.77, *p* = 0.476, $\eta_{G}^{2}=$ 0.010) and no distance × size interaction (*F*_8,88_ = 0.83 *p* =0.575, $\eta_{G}^{2}=$ 0.033).

These results clearly show that the neighbourhood of interaction does not have a fixed radius of 4 m, for pedestrians may be influenced by neighbours at three times that distance—if they are fully visible. This finding suggests that there may be two decay processes at work: a gradual decay to visible neighbours, and a more rapid decay within a partially occluded crowd.

## 4. Experiment 2: the double-decay hypothesis

**Figure 3.**
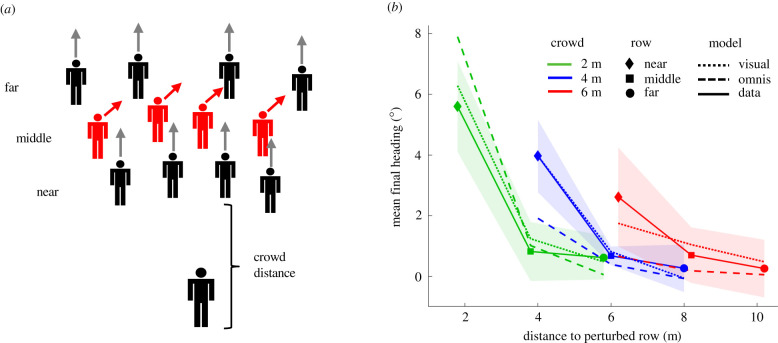
Experiment 2: double-decay hypothesis. (*a*) Schematic of virtual crowd, illustrating a rightward heading perturbation of the middle row. (*b*) Results: mean final heading as a function of distance to the perturbed row (symbols), for each crowd distance (curves). Solid curves represent human data, dotted curves the visual model and dashed curves the omniscient model. Shaded regions represent 95% confidence intervals for the human data. (Models were not intended to reproduce gait oscillations, so their variable error is small and not represented.) (Online version in colour.)

The second experiment tested this ‘double-decay' hypothesis, specifically that there are two decay processes which depend on distance. We manipulated a virtual crowd of 12 neighbours, randomly positioned in three rows spaced 2 m apart (figure 3*a*). To check the decay rate to fully visible neighbours, we varied the distance of the near row (2, 4 or 6 m). To probe the decay rate within the crowd, we selectively perturbed the near, middle or far row, so all neighbours in one row turned in the same direction (±10°). Farther neighbours were thus dynamically occluded by nearer neighbours.

### (a) Results

Final heading is plotted as a function of distance to the perturbed row in figure 3*b*, where each curve represents a crowd distance (i.e. to the near row). Two decay rates are immediately apparent. First, the heading response decreases with the distance of the crowd (*F*_2,18_ = 26.68, *p* < 0.001, $\eta_{G}^{2}=$ 0.229). In particular, the response to perturbations of the near row (diamonds) decays gradually with distance (simple effect test, *F*_2,18_ = 48.46, *p* < 0.001), replicating experiment 1. Linear extrapolation suggests an interaction range of at least 9 m (*y* = −0.81*x* + 7.33, *r*_2_ = −0.99). The decay rate (slope) is slightly steeper, and responses are weaker (intercept), than in experiment 1, owing to the presence of unperturbed neighbours, yielding a shorter interaction range.

Second, for each curve, the heading response decreases more rapidly within the crowd (*F*_2,18_ = 86.98, *p* < 0.001, $\eta_{G}^{2}=$ 0.760). It drops steeply from the near row to the middle row (*t*_9_ = 10.82, *p* < 0.001, Cohen's *d* = 3.42) and the far row (*t*_9_ = 11.95, *p* < 0.001, Cohen's *d* = 3.77). This finding implies that dynamic occlusion by near neighbours weakened responses to the middle and far rows, almost to the floor of zero.

The evidence thus reveals that the neighbourhood of interaction results from two decay processes. We propose, first, that the gradual decay to visible neighbours follows from Euclid's Law of perspective, which states that the visual angle subtended by an object (or motion) with frontal extent *x* diminishes with distance *z* as tan^−1^(*x*/*z*). Note that this predicts a larger range of interaction than simple linear extrapolation. Second, the more rapid decay within the crowd is owing to the additional effect of occlusion. These findings led us to formulate a new visual model.

## 5. Visual model

To build a visual model of collective motion from the bottom up, we begin with the visual coupling between a pedestrian and a single neighbour [38,42,43].

### (a) Heading control

**Figure 4.**
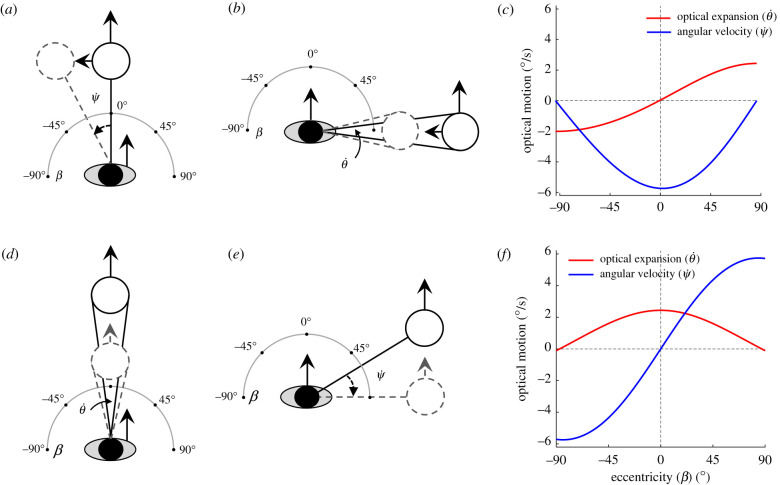
Visual information for control of heading (top) and speed (bottom). See text for explanation. Oval = pedestrian, open circle = neighbour, $\;\dot{\;\psi }=\mathrm{angular}\;\mathrm{velocity}$, $\;\dot{\theta }=\mathrm{expansion}\;\mathrm{rate}$ and *β* = eccentricity. Optical motions are computed for a neighbour with diameter = 0.4 m, distance = 1 m, relative speed = −1 m s^−1^ leftward (*c*) or −0.1 m s^−1^ backward (*f*). (Online version in colour.)

Consider a pedestrian following a neighbour who turns left (figure 4, top row). If the neighbour is directly ahead (eccentricity of *β* = 0°, with positive angles to the right and negative angles to the left), this generates a leftward angular velocity (negative $\;\dot{\;\psi }$) in the pedestrian's field of view (figure 4*a*). Cancelling $\dot{\;\psi }$ would cause the pedestrian to steer left and approximately match the neighbour's heading. On the other hand, if the neighbour is to the pedestrian's right (*β* = 90°), a left turn generates an optical expansion ($\dot{\theta }$) in the field of view (figure 4*b*). In this case, cancelling $\;\dot{\theta }$ would cause the pedestrian to steer left and match the neighbour's heading. Critically, optical velocities ($\;\dot{\;\psi },\dot{\theta }$) decrease with neighbour distance in accordance with Euclid's Law.

These two optical variables thus trade off as a function of the neighbour's eccentricity (figure 4*c*). For a left turn, angular velocity $\;\dot{\;\psi }$ (blue curve) is a cosine function of eccentricity with a minimum (leftward motion) at *β* = 90°, whereas expansion rate $\;\dot{\theta }$ (red curve) is a sine function with a minimum (contraction) at *β* = −90° and a maximum (expansion) at *β* = 90°. For a right turn, these functions flip about the horizontal axis.

The visual coupling for controlling heading (*ϕ*) can thus be formalized as a second-order control law,

5.1 $$\;{\ddot{\;\phi }}_{p}=-\;c_{1}(\cos\beta_{i})\dot{\psi_{i}}+c_{2}(\sin\beta_{i})\;{\dot{\theta }}_{i},$$

in which pedestrian *p* steers (angular acceleration $\ddot{\phi }$) so as to cancel the combined angular velocity ($\;\dot{\;\psi }$) and expansion rate ($\dot{\theta }$) of neighbour *i*. Their dependence on *β* acts as a filter so that steering is influenced by combinations of variables that correspond to a turning neighbour at that eccentricity. The free parameters (*c*_1_ = 14.38, *c*_2_ = 59.71) were fitted to our previous data on pedestrian following [39,42] and held constant.

### (b) Speed control

The control of walking speed is complementary to the control of heading (figure 4, bottom row). If a neighbour directly ahead (*β* = 0°) slows down, this generates an optical expansion ($\dot{\theta }$) in the pedestrian's field of view (figure 4*d*). Cancelling the expansion would cause the pedestrian to decelerate and approximately match the neighbour's speed. However, if a neighbour to the pedestrian's right (*β* = 90°) slows down, this generates a rightward angular velocity (positive $\;\dot{\;\psi }$) in the field of view (figure 4*e*); cancelling it would also lead the pedestrian to decelerate to the neighbour's speed. These two optical variables again trade off as a function of eccentricity, but with the opposite sine and cosine functions (figure 4*f*). If the neighbour speeds up, the curves flip about the horizontal axis.

The visual coupling for control of radial speed ($\dot{r}$) is thus based on the same two optical variables as in equation (5.1), but the sine and cosine functions are reversed:

5.2 $${\ddot{r}}_{p}=-\;c_{3}(\sin\beta_{i})\dot{\psi_{i}}-c_{4}(\cos\beta_{i})\;{\dot{\theta }}_{i}.$$

Pedestrian *p* thus linearly accelerates or decelerates ($\ddot{r}$) so as to cancel the combined angular velocity ($\;\dot{\;\psi }$) and expansion rate ($\dot{\theta }$) of neighbour *i*. But now *p*'s speed is influenced by combinations of variables that correspond to a neighbour changing speed at a given eccentricity. The free parameters (*c*_3_ = 0.18, *c*_4_ = 0.72) were fitted to our data on pedestrian following [39,42] and held fixed. To normalize for variation in neighbour size, the relative rate of expansion ($\dot{\theta }/\theta$) can be substituted for expansion rate ($\dot{\theta }$) [43].

### (c) Collective motion

To formulate a model of collective motion, we substitute the visual control laws for local interactions (equations (5.1) and (5.2)) into a neighbourhood function that averages the influences of multiple neighbours (refer to the electronic supplementary material, equation S1):

5.3 $${\ddot{\phi }}_{p}=\frac{1}{n}\overset{n}{\underset{i=1}{\sum }}v_{i}[-\;c_{1}(\cos\beta_{i})\dot{\psi_{i}}+c_{2}(\sin\beta_{i}){\;\dot{\theta }}_{i}]$$

and

5.4 $${\ddot{r}}_{p}=\frac{1}{n}\overset{n}{\underset{i=1}{\sum }}v_{i}[-\;c_{3}(\sin\beta_{i})\dot{\psi_{i}}-c_{4}(\cos\beta_{i}){\;\dot{\theta }}_{i}].$$

Pedestrian *p*'s heading and speed are thus controlled by cancelling the mean angular velocity ($\;{\dot{\;\psi }}_{i}$) and rate of expansion (${\dot{\theta }}_{i}$) of all visible neighbours (*i* = 1 … *n*), depending on their eccentricities (*β_i_*). The field of view is centred on the heading direction, as people tend to face in the direction they are walking [45].

Partial occlusion is incorporated by weighting each neighbour in proportion to their visibility, *v_i_*, which ranges from 0 (fully occluded) to 1 (fully visible). If the visibility falls below a threshold value (*v_t_* = 0.15), *v_i_* is set to 0; thus, *n* is the number of visible neighbours above threshold. Importantly, the occluded region behind a neighbour grows with distance, so the visibility of far neighbours tends to decrease with their separation in depth from near neighbours (figure 1*b*). Consequently, the range of interaction depends on the crowd's *opacity* [46] and is limited by the complete occlusion of far neighbours.

Basic properties of physics-inspired models fall out naturally from the visual model. First, cancelling optical expansion yields collision avoidance without an explicit ‘repulsion' force. Second, cancelling optical contraction maintains group cohesion without an explicit ‘attraction' force. Third, cancelling the combined angular velocity and expansion/contraction generates collective motion without an explicit ‘alignment' rule. Finally, the laws of optics account for the form of the neighbourhood without an explicit decay function: Euclid's Law explains the gradual decay of influence to visible neighbours, and the added effect of occlusion explains the more rapid decay within a crowd.

## 6. Model simulations

We tested the visual model (equations (5.3)–(5.4)) by predicting human trajectories in virtual crowd experiments and real crowd data and compared the results to our previous omniscient model [8]. We find that the visual model outperforms the omniscient model (and a model based on optical motion without occlusion, see the electronic supplementary material) and generalizes to real crowds.

To simulate each experimental trial, the models were initialized with the participant's position, heading and speed 2 s before the perturbation. For the omniscient model, the input on each time step was the position, heading and speed of all virtual neighbours in the HMD's 90° field of view on that trial. For the visual model, the input was the angular velocity, expansion rate, eccentricity and visibility of the same neighbours, calculated from their positions on each time step. The output of both models was the position, heading and speed of the simulated agent on the next time step, represented as time series for each trial. As a measure of model performance, we computed the mean position error (ME) or root mean squared error (RMSE) in heading and speed between each participant's mean time series in each condition and the corresponding mean time series for the model.

### (a) Simulating experiment 2

First, we simulated the double-decay experiment. For the omniscient model, we added a gradual exponential term to the decay function (electronic supplementary material, equation S4), estimated from the data. Because crowd speed was not manipulated in this experiment, we used the participant's recorded walking speed as input to the omniscient model. Mean final heading for the two models is plotted in figure 3*b*, together with the human results. Although both models are close to the 95% confidence intervals (CIs) for the human data (shaded regions), the visual model (dotted curves) lies entirely within them.

Over the whole time series, the mean heading error for the visual model (RMSE_V_ = 2.47°) was significantly smaller than that for the omniscient model (RMSE_O_ = 3.45°) (*t*_9_ = 14.48, *p* < 0.001, Cohen's *d* = 1.460); a Bayes factor (BF) indicated decisive evidence for the alternative hypothesis (BF_10_ ≫ 100). The mean position error for the visual model (ME_V_ = 0.241 m) was also smaller than that for the omniscient model (ME_O_ = 0.309 m) (*t*_9_ = 8.46, *p* < .001, Cohen's *d* = 0.294), decisive evidence (BF_10_ ≫ 100).

In summary, the visual model predicted the range of interaction better than the omniscient model because the decay rate is not a constant function of distance but depends on the amount of occlusion. The visual model thus accounts for the form of the neighbourhood without an explicit decay function.

### (b) Re-simulating Rio *et al.* [8]

**Figure 5.**
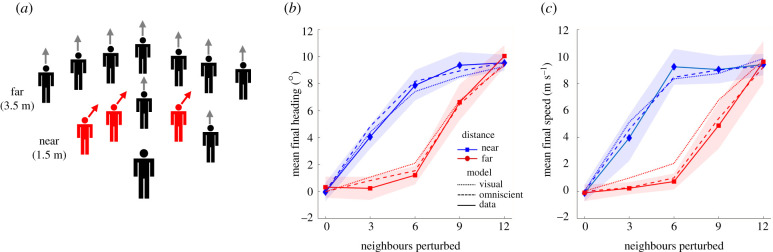
Rio *et al.*'s [8] experiment 2. (*a*) Schematic of virtual crowd (12 neighbours). A subset of neighbours (0–12) was perturbed, predominantly in the near or the far row. (*b*) Results for heading perturbation: mean final heading as a function of the number of perturbed neighbours, for each row (curves). (*c*) Results for speed perturbation: mean final speed as a function of same. Solid curves represent human data, dotted curves the visual model and dashed curves the omniscient model. Shaded regions represent 95% confidence intervals for the human data. (Modified from [8], with permission.) (Online version in colour.)

As a further test of the models, we re-simulated Rio *et al*.'s [8] experiment 2, which perturbed heading or speed and manipulated the number and distance of perturbed neighbours (figure 5*a*). The virtual crowd contained five neighbours in the near row (1.5 m) and seven in the far row (3.5 m). On each trial, a subset of neighbours, predominantly in one row, either turned ±10° or changed speed by ±0.3 m s^−1^ (from 1.0 m s^−1^). Mean final heading and mean final speed appear in figure 5*b,c* (solid curves). Responses were larger when near neighbours were perturbed (top curve) than when far neighbours were perturbed (bottom curve), reflecting the decay of influence with distance.

Simulations of the visual model (dotted curves) and the original omniscient model (dashed curves; electronic supplementary material, equations S1–S3) appear in figure 5*b*,*c*. Both are close to the human data (solid curves), falling within the 95% CIs in nearly all conditions. Over time, the mean heading error was significantly smaller for the visual model (RMSE_V_ = 1.97°, RMSE_O_ = 2.08°), (*t*_9_ = 6.94, *p* < 0.001, Cohen's *d* = 0.871, BF_10_ > 100), although there was no difference in the mean speed error (RMSE_V_ = 0.0627 m s^−1^, RMSE_O_ = 0.0640 m s^−1^) (*t*_9_ = 1.15, *p* = 0.281, Cohen's *d* = 0.208; BF_01_ = 1.91, anecdotal evidence for the null hypothesis) or the mean position error (ME_V_ = 0.193 m, ME_O_ = 0.199 m) (*t*_9_ = 1.112, *p* = 0.295, Cohen's *d* = 0.082; BF_01_ = 1.96, anecdotal). Both models thus capture the human data quite well, although the visual model performs better on heading.

The comparatively good performance of the omniscient model in this experiment stems from the fact that the decay function was originally fitted to human swarms that had nearest-neighbour distances (1–3 m) and densities similar to those of the virtual crowd. However, this empirical decay term did not generalize to larger distances in the double-decay experiment, whereas the visual model did so.

In summary, the visual model accounts for Rio *et al*.'s [8] experiment as well or better than the omniscient model. Whereas the latter assumes physical variables as input, the former is based on optical variables available to an embedded pedestrian: far neighbours exert less influence because they have lower optical velocities and are partially occluded by near neighbours.

### (c) Human swarm simulations

To test whether our findings for virtual crowds apply to real crowds, we simulated walking trajectories in previously recorded data on human ‘swarms' [8]. We attempted to predict the trajectory of an individual pedestrian from the movements of their neighbours using both models.

Three different groups of participants (*n* = 10, 16 and 20) were instructed to walk about a large tracking area (14 × 20 m), veering left and right while staying together as a group, for a total of twelve 2 min trials. Head-mounted markers were recorded with 16 motion-capture cameras (Qualisys) at 60 Hz, and time series of head position, heading and speed were computed as before. We identified thirty 10 s segments of data in which ≥75% of the participants were continuously tracked. For each segment, we simulated a focal participant at the back of the group and treated the tracked neighbours as input. For the visual model, we computed optical variables from neighbour positions and velocities. The omniscient model used the original decay function (electronic supplemenatry material, equation S3).

**Figure 6.**
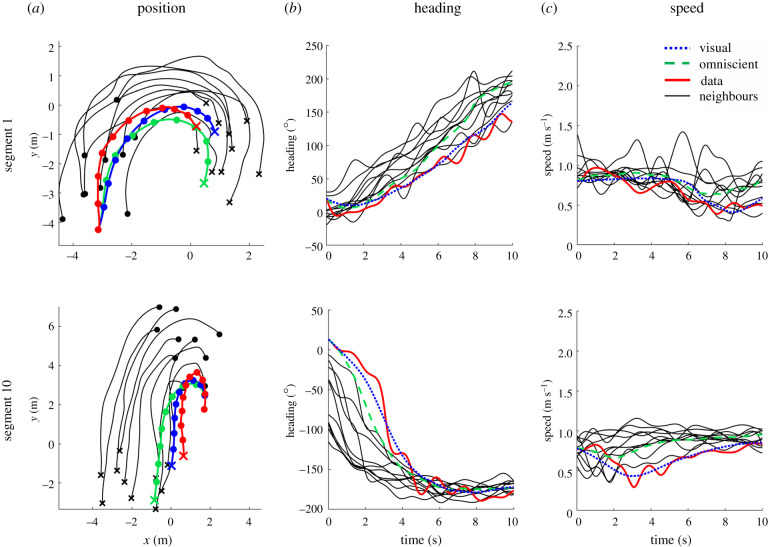
Sample segments (10 s) from the human swarm, with focal participant (red solid curve) and simulations of visual model (blue dots) and omniscient model (green dashes). (*a*) Traces of position over time (segment 1: ME_V_ = 0.379 m, ME_O_ = 0.818 m; segment 10: ME_V_ = 0.275 m, ME_O_ = 1.389 m). (*b*) Time series of heading (segment 1: RMSE_V_ = 10.67°, RMSE_O_ = 32.88°; segment 10: RMSE_V_ = 11.81°, RMSE_O_ = 23.62°). (*c*) Time series of speed (segment 1: RMSE_V_ = 0.187 m s^−1^, RMSE_O_ = 0.162 m s^−1^; segment 10: RMSE_V_ = 0.157 m s^−1^, RMSE_O_ =0.178 m s^−1^). Thin grey curves = neighbours; x = final positions, filled circles at 1 s intervals. Note that errors are higher than those in virtual crowds because they are computed on single trials rather than mean time series and thus reflect gait oscillations and tracking errors. (Online version in colour.)

Two segments of simulated swarm data appear in figure 6. The heading time series (column (*b*)) for the focal participant (solid curve) is more closely captured by the visual model (dots) than the omniscient model (dashes) in both segments, whereas the speed time series (column (*c*)) is better approximated by the omniscient model in segment 1 (top) and the visual model in segment 10 (bottom). Over all 30 segments, the mean heading error was significantly lower for the visual model (RMSE_V_ = 15.0°) than the omniscient model (RMSE_O_ = 22.9°) (*t*_29_ = 4.48, *p* < 0.001, Cohen's *d* = 0.806; BF_10_ > 100, decisive evidence), as was the mean position error (ME_V_ = 0.60 m, ME_O_ = 0.80 m) (*t*_29_ = 2.21, *p* < 0.05, Cohen's *d* = 0.338; BF_10_ = 1.60 anecdotal evidence). On the other hand, the mean speed error was significantly lower for the omniscient model (RMSE_V_ = 0.224 m s^−1^, RMSE_O_ = 0.146 m s^−1^) (*t*_29_ = 6.83 *p* < 0.001, Cohen's *d* = 1.198; BF_10_ ≫ 100, decisive evidence); we consider this result in §7.

The visual model thus accounts for individual heading and position in real crowd data better than the omniscient model, even though the latter's decay term was fitted to a sample of the same data. We attribute this advantage largely to the effect of occlusion. Whereas the omniscient model approximates the decay with distance using a fixed exponential function, the visual model incorporates dynamic occlusion and is thus sensitive to changes in visibility over time.

## 7. Discussion

Nearly all microscopic models of collective motion in humans and animals attribute local interactions to hypothetical rules or forces and assume physical variables as input. In this article, we developed a visual model of human ‘flocking' grounded in the visual coupling with optical variables as input. In contrast to previous phenomenological models, the visual model explains basic properties of interaction as natural consequences of the laws of optics.

First, *social forces* and *rules of engagement* are reduced to optical variables that control an individual's heading and speed. In place of explicit ‘repulsion' and ‘attraction' forces, collision avoidance results from cancelling optical expansion, and group cohesion is maintained by cancelling optical contraction. Instead of an explicit ‘alignment' rule, collective motion emerges from cancelling the combined expansion/contraction and angular velocity of neighbours. The visual coupling thus acts functionally like a force or 'optical push' [47].

Second, the *neighbourhood of interaction* is explained by the laws of optics, without an explicit distance term. The gradual decay to visible neighbours in the field of view follows from Euclid's Law, the diminution of optical velocity with distance. The more rapid decay within a crowd follows from the added effect of visual occlusion, which grows with the separation in depth between near and far neighbours. Consequently, the neighbourhood range and number of neighbours *n* are not determined by a fixed distance but vary with crowd opacity.

The visual model thus predicts that the effective neighbourhood depends on crowd density, which we confirmed in related experiments [48]. In dense human crowds (1–2 m apart), complete opacity can occur by a range of 5 m. Starlings appear to adjust flock density to maintain ‘marginal opacity' such that individual birds can see through the entire flock [46]. The range of interaction might also be limited by a visual detection threshold for optical motion. However, adding a motion threshold in our simulations did not improve the fit to the data, perhaps because it was superseded by occlusion.

Nearly all physics-inspired models assume the principle of superposition, according to which the response to a group is the linear combination of independent responses to each neighbour. But, superposition is invalidated by the facts of visual occlusion: because the influence of far neighbours depends on the positions of near neighbours, the response to the former is not independent of the latter. While this may be computationally inconvenient, visual occlusion has large effects on local interactions and should be incorporated into future models [30,31].

Note that Euclid's Law predicts an asymmetry in the pedestrian's response. Given a neighbour an initial distance ahead, if they slow down, their distance decreases, whereas if they speed up, their distance increases. Consequently, the rate of expansion is greater than the rate of contraction for the same speed change. This effect explains an asymmetric speed response we previously observed in pedestrian following [38,43].

The visual model generally outperforms the omniscient model, although they were quite similar in our re-simulation of Rio *et al*.'s [8] experiment. That result is attributable to the fact that the omniscient model approximates the decay with distance using an exponential function that was fitted to human swarms with a similar distance and density to the virtual crowd. However, this fixed decay term did not generalize to other crowd distances in experiment 2, whereas the visual model did so. The visual model thus not only explains the form of the neighbourhood but generalizes to new conditions without re-parameterization.

We noted a limitation of the current visual model when we were simulating the human swarm data. In five additional segments, the front of the crowd executed a 180° hairpin turn and walked back towards the focal participant, generating rapid expansion in the field of view. Human participants kept walking forwards, but the visual model responded by slowing down and backing up to cancel the optical expansion. Similar but less extreme responses to U-turns may explain the larger speed error for the visual model reported above. Clearly, the model needs to distinguish neighbours that should be followed from obstacles that should be avoided, which may be as straightforward as discriminating the front and back of other pedestrians.

Our findings suggest that characteristic patterns of collective motion in different species might result from a reliance on different sensory variables. Humans cancel optical velocities, which yields collective motion despite variation in neighbour distance, density and size. By contrast, holding the visual angles of near neighbours at a particular value would yield fish schools with a preferred spatial scale, whereas maintaining neighbours in particular visual directions would yield bird flocks with a preferred spatial structure.

In summary, we conclude that the local interactions underlying collective motion have a lawful basis in the visual coupling between neighbours. In recent multi-agent simulations, we have also shown that the visual model generates emergent collective motion, and a report is in preparation.

## Data Availability

Data and computer code are available from the Brown Digital Repository: https://doi.org/10.26300/r4c3-dq82 [49].
